# Determinants of Symptomatic Vulvovaginal Candidiasis among Human Immunodeficiency Virus Type 1 Infected Women in Rural KwaZulu-Natal, South Africa

**DOI:** 10.1155/2014/387070

**Published:** 2014-04-09

**Authors:** Teke Apalata, William H. Carr, Willem A. Sturm, Benjamin Longo-Mbenza, Prashini Moodley

**Affiliations:** ^1^Department of Infection Prevention and Control and Medical Microbiology, School of Laboratory Medicine and Medical Sciences, College of Health Sciences, University of KwaZulu-Natal, Private Bag 7, Congella, Durban 4013, South Africa; ^2^Department of Medical Microbiology, Faculty of Health Sciences, Walter Sisulu University, Private Bag X1, Mthatha, Eastern Cape 5117, South Africa; ^3^HIV Pathogenesis Programme (HPP), Department of Paediatrics and Child Health, Nelson R Mandela School of Medicine, University of KwaZulu-Natal, Private Bag 7, Congella, Durban 4013, South Africa; ^4^Department of Biology, Medgar Evers College, City University of New York, Brooklyn, NY 11225, USA

## Abstract

*Introduction*. We sought to determine the association between HIV-induced immunosuppression, virologic correlates, and vulvovaginal candidiasis (VVC). *Methods*. This is a retrospective cohort study, where HIV infected and uninfected women were studied with VVC being the primary outcome. Ninety-seven HIV-infected and 101 HIV-uninfected women were enrolled between June and December 2011. Cases of VVC were confirmed. HIV RNA load was determined by RT-PCR and CD4 counts were obtained from medical records. *Results*. Fifty-two of 97 (53.6%) HIV-infected and 38/101 (37.6%) HIV-uninfected women were diagnosed with VVC (*P* = 0.032). The relative risk for VVC amongst HIV-infected patients was 1.53 (95% CI: 1.04–2 *P* = 0.024). Cases of VVC increased at CD4+ T cell count below 200 cells/mm^3^ (*P* < 0.0001) and plasma HIV RNA load above 10 000 copies/mL (*P* < 0.0001). VVC was associated with increased genital shedding of HIV (*P* = 0.002), and there was a linear correlation between plasma HIV load and genital HIV shedding (*r* = 0.540; *R* ^2^ = 0.292; *P* < 0.0001). Women on HAART were 4-fold less likely (*P* = 0.029) to develop VVC. *Conclusion*. CD4 counts below 200 cells/mm^3^ and plasma HIV loads ≥10 000 copies/mL were significantly associated with VVC.

## 1. Introduction

Symptomatic vulvovaginal candidiasis (VVC) is caused by a number of species belonging to the genus* Candida* which are commensal fungi of the gastrointestinal tract and vagina.* Candida albicans* has been reported as the cause of symptomatic VVC in 85%–95% of cases, whilst* C. glabrata* represents the most common cause of nonalbicans candida vaginitis [1].

Whilst 75% of healthy women develop symptomatic VVC at least once during their reproductive age, it is estimated that 5%–8% of women develop recurrent vulvovaginal candidiasis (RVVC). The latter is defined as the occurrence of four or more episodes of symptomatic VVC per year [1].

In HIV infected patients, whilst oroesophageal candidiasis is known to appear at any time during the course of progression of HIV infection, symptomatic VVC develops significantly later [2]. It has been shown that oroesophageal candidiasis occurs as a result of the loss of mucosal and systemic cell-mediated immunity, while animal studies, in vitro experiments, and clinical trials indicate that systemic cell-mediated immunity does not play a protective role against candida vaginitis [3–5].

Cross-sectional studies of women attending antenatal clinics and family planning clinics in South Africa over the past 20 years showed a weighted average prevalence of 26% for symptomatic VVC [6–8]. Among these studies, many have been conducted in KwaZulu-Natal where there is a high prevalence of HIV in South Africa. Data from the national population-based survey conducted in 2008 estimated HIV prevalence in young people aged 15–24 years to be 15.3% (95% CI 11.8–19.7) in the province of KwaZulu-Natal compared to the national estimate of 8.7% (95% CI 7.2–10.4) [9].

Ramjee et al. reported the prevalence of 36.1% and 45.7% for symptomatic VVC, respectively, among HIV negative and HIV positive sex workers in KwaZulu-Natal [7]. In 2011, during a nested control analysis from a prospective trial of 1 404 women (716 HIV infected compared with 688 HIV noninfected) over a 2-year period in KwaZulu-Natal, Sebitloane, Moodley, and Esterhuizen reported on symptomatic VVC as being significantly more prevalent among more HIV infected than uninfected women (39.2% versus 30%; RR = 1.31 (1.08–1.59); *P* = 0.006) [8].

The aim of this study was to determine predictors of symptomatic VVC in HIV infected women.

## 2. Materials and Methods

### 2.1. Patients and Sampling Strategy

**Figure 1:**
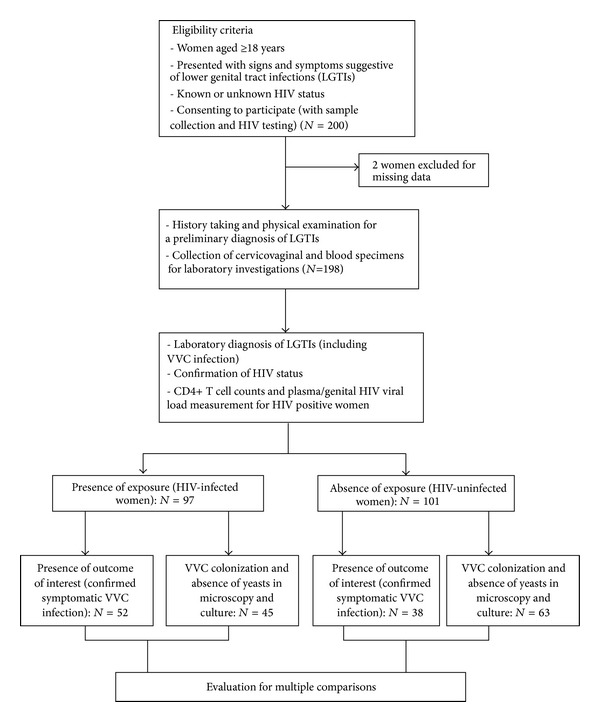
Selection criteria and characterization of the study population by HIV groups.

This is a retrospective cohort study design. Consecutive consenting female patients attending Umlazi D clinic, a primary healthcare facility in KwaZulu-Natal, were recruited between June 2011 and December 2011. Presentation with lower genital tract signs and symptoms was one of the eligibility criteria as depicted in Figure 1.

A standardized questionnaire was used to collect patient demographic information, history of STIs and treatment thereof over the past 3 months, condom utilization behaviour, and prior knowledge of HIV serostatus. HIV positive women were further questioned about receiving HAART, and this was confirmed by a review of the medical records.

CD4 counts were obtained from patients' medical records and were not older than 3 months. Blood and genital secretions for measurement of HIV viral loads were collected from patients at recruitment.

Patients were invited to participate in the study if they had signs and symptoms of vaginal discharge syndrome. All patients were treated syndromically as per standard of care STI treatment in South Africa. The study was approved by the Biomedical Research Ethics Committee of the University of KwaZulu-Natal (Ref. BE 224/11). Consent forms were signed by all participants and confidentiality was maintained throughout the study.

### 2.2. Specimen Collection and Processing

One cervical and two vaginal swabs (Probetec swabs, Becton Dickinson, Sparks, Maryland, USA) were used. The two vaginal swabs collected material from the anterior and posterior fornix, respectively. A smear was made on a glass slide using the latter, and the former as well as the cervical swab was placed in a sterile dry container. A vaginal tampon (8 Ks, Tampax Regular Compak) was thereafter inserted into the vagina and removed after 3 minutes. This was placed in a sterile container with 10 mL of phosphate buffered saline (PBS; Oxoid Limited Basingstoke, Hampshire, UK) (pH = 6.9). Blood samples were collected into ethylenediaminetetraacetic acid (EDTA) and non-EDTA containing tubes.

All specimens were kept in a cooler box with ice awaiting transportation (within 4 hours) to the Infection Prevention and Control, Nelson R Mandela School of Medicine, UKZN.

The anterior fornix vaginal swab was used to inoculate Sabouraud dextrose agar with chloramphenicol (BBL Becton Dickinson) and incubated at 29°C for 48 h. The relative vaginal fungal burden was estimated as previously described [10].

The smear was stained using Gram's method and evaluated by two independent microscopists for bacterial vaginosis (BV) using Nugent's score [11]. Discrepancies were evaluated by an independent medical microbiologist.

The cervical swab was used for the detection of* Chlamydia trachomatis* and* Neisseria gonorrhoeae* using a strand displacement amplification technology (Becton Dickinson Probetec Assays, Sparks, Maryland, USA) method as previously described [12]. DNA of* Mycoplama genitalium*,* Trichomonas vaginalis*, and herpes simplex virus type 2 was obtained using QIAamp DNA mini kits (Qiagen Ltd., Chatsworth, CA) [12]. An in-house PCR method for amplification was used as previously described [12–14]. The amplified PCR product was analyzed by gel electrophoresis.

Vaginal fluid was expressed from vaginal tampon using an autoclaved wooden tongue depressor and filtered through a 0.22 *μ*m Costar Spin-X cellulose acetate filter membranes (Sigma) and used for quantifying HIV viral load in genital secretions.

### 2.3. Diagnostic Criteria of Symptomatic Vulvovaginal Candidiasis and VVC Colonization

The diagnosis of symptomatic VVC was based on a combination of clinical and laboratory criteria (evidence level III, recommendation grade B) as described by the European (IUSTI/WHO) guideline on the management of vaginal discharge, 2011 [10].

Symptoms suggestive of symptomatic VVC included vulval pruritus/itching, vulval soreness, superficial dyspareunia, and/or malodorous vaginal discharge. Signs included vulval erythema, vulval oedema, fissures, excoriation, or thick curdy vaginal discharge.

In addition to the self-reported above symptoms and observation of signs suggestive of VVC in physical examination, cases of symptomatic VVC were confirmed if one of the following criteria was fulfilled: (i) a positive Gram-stain preparation with budding yeasts, pseudohyphae, and/or hyphal forms; (ii) positive culture with either moderate (10–99 colonies per plate) or heavy candida growth (>100 colonies per plate).

Participants without symptomatic VVC were defined as (i) patients whose genital specimens had a negative microscopy result for yeasts, pseudohyphae, and/or hyphal forms of candida together with negative culture; (ii) patients whose genital specimens had a negative microscopy result for yeasts, pseudohyphae, and/or hyphal forms of candida together with light candida growth (<10 colonies per plate). The latter was considered to indicate vaginal* candida* colonization rather than infection.

### 2.4. HIV Testing and Definitions of HIV-Induced Immunosuppression

Initial HIV test was performed in the clinic by a trained research nurse on blood using the HIV rapid test Determine HIV-1/2/O (Abbott Laboratories, Abbott Park, IL) following voluntary counseling and testing. The diagnosis of HIV negative with an appointment for a further rapid testing 3 months later was given to the patient following a negative initial test. Positive samples were transported in the Infection Prevention and Control research laboratory at the Nelson R Mandela School of Medicine, UKZN, and were subsequently retested by a medical technologist using a second HIV rapid test SmartCheck test (World Diagnostics Inc., USA). A diagnosis of HIV positive was reported to the participants only if both rapid tests were positive. Samples that showed discordant results were further evaluated with a third rapid test Uni-Gold Recombigen HIV (Trinity Biotech PLC, USA), and only two positive test results were interpreted as a positive diagnosis for HIV.

As part of the routine management of the patients in the clinic, all HIV-infected patients benefited directly from CD4+ T cell count measurements, and CD4+ T cell counts used in this study were obtained from patients' medical records and were not older than 3 months. However, at the time patients were evaluated for this research, HIV-1 RNA was measured from the plasma and cell-free fraction of vaginal secretions using Nuclisens Easyq HIV-1 assay v2.0 (BioMerieux, Lyon, France) with a lowest detection limit of 20 copies/mL.

Absolute values of CD4+ T cell counts (cells/mm^3^) were used to determine the degree or severity of immunocompromise following the World Health Organization (WHO) immunological staging criteria: CD4 levels <200/mm^3^ (severe immunosuppression), CD4 levels 200–349/mm^3^ (advanced immunosuppression), and CD4 levels 350–499/mm^3^ (mild immunosuppression) [15].

In addition, virological correlates (plasma HIV viral loads) were also measured since they are potentially useful markers of disease progression (although not reported to be consistent markers) and have been shown by others to increase across progressive stages of HIV infection [16]. For the purpose of this study, plasma viremia was classified as <20 copies/mL (below detectable level of the used test), 20–9999 copies/mL, and ≥10 000 copies/mL. We also log transformed HIV loads for improved symmetry and included them in multivariate logistic regression models as continuous values.

### 2.5. Statistical Analyses

Data analysis was performed using SPSS statistical software version 21.0 (SPSS Inc., Chicago, IL). Data were expressed as means ± standard deviation (SD) for the continuous variables and proportions (percentages) for the categorical variables. Student's *t*-test was performed to assess differences between two means and ANOVA between groups. When data were not normally distributed, the Mann-Whitney *U* test was used. Either chi-square test with and without trend or Fischer's exact test was used to test the degree of association of categorical variables.

Multiple logistic regression models were used to evaluate the prediction capacity of each independent variable in the occurrence of the expected condition. Unadjusted odds ratios (ORs) were initially calculated to screen for inclusion in multivariate models; variables that exhibited at least moderate association (*P* < 0.20) with the outcome were considered for inclusion in the final models. Multivariate ORs (95% CI) were computed after adjusting for confounding univariate factors. All tests were two-sided and a *P* value of <0.05 was considered significant.

## 3. Results

Of the 200 patients enrolled with vaginal discharge syndrome (VDS), 2 were excluded for missing results. Of the 198 participants, 97 were HIV-infected and 101were HIV-uninfected. This was a simple coincidence that the first 200 women who met the eligibility criteria comprised of approximately equal numbers of HIV positive and HIV negative.

From the 97 HIV-infected women, 52 cases of symptomatic VVC were diagnosed (53.6%), while 38 cases of symptomatic VVC were diagnosed among the 101 HIV-uninfected women (37.6%) (*P* = 0.032). The relative risk value for symptomatic VVC amongst HIV-infected patients was 1.53 (95% CI: 1.04–2 *P* = 0.024).  Table 1 depicts the baseline characteristics of the study population.

In HIV-infected women, univariate analysis (Table 2) showed that women's vaginal flora with Nugent's score <7 (*P* = 0.01), plasma HIV viral loads ≥10 000 copies/mL (*P* < 0.0001), genital HIV viral loads ≥10 000 copies/mL (*P* = 0.002), CD4 levels <200 cells/mm^3^ (*P* < 0.0001), and absence of HAART (*P* < 0.001) were significantly associated with symptomatic VVC. There was a linear correlation between plasma HIV viral load and genital HIV shedding (*r* = 0.540; *R* ^2^ = 0.292; *Y* = 1.51 + 0.71∗*X*; *P* < 0.0001).

Findings have shown that HIV viral loads increased across progressive stages of HIV-induced immunocompromise: CD4 count <200 cells/mm^3^, between 200–349 cells/mm^3^ and ≥350 cells/mm^3^ significantly correlated with the mean plasma HIV load of 4.43  (4.1–4.9)Log_10_, 2.24  (2–2.5)Log_10_, and 1.3Log_10_, respectively (*P* < 0.0001), and also with the mean genital HIV load of 2.51  (2.1–2.9)Log_10_, 1.63  (1.4–1.9)Log_10_, and 1.66  (1.3–2)Log_10_, respectively (*P* < 0.001).

Plasma HIV viral load and genital HIV viral load significantly varied among HAART groups. Higher mean of Log_10_ HIV viral load values was shown in the plasma (3.41  (2.8–4)Log_10_) of HIV-infected women antiretroviral therapy (ART) naïve as compared to patients on HAART (2.68  (2.34–3)Log_10_) (*P* = 0.014). Similarly, higher mean of Log_10_ HIV viral load values was also observed in the genital tracts (2.61  (2.1–3.10)Log_10_) of HIV-infected women antiretroviral therapy (ART) naïve as compared to patients receiving HAART (1.67  (1.4–1.9)Log_10_) (*P* < 0.0001).

Bacterial vaginosis was frequent among HIV infected women in the absence of symptomatic VVC (*P* = 0.01) (Table 2). There was also a significant association between bacterial vaginosis and higher levels of HIV RNA in the plasma. Nugent scores between 0–3, 4–6, and 7–10 were associated with mean plasma HIV loads of 1.9Log_10_, 2.45Log_10_, and 3.20Log_10_, respectively (ANOVA, *P* = 0.006). However, higher levels of genital HIV RNA were not significantly associated with Nugent score categories (ANOVA, *P* = 0.062).

Findings showed a negative but not significant (Spearman's Rho = −0.101; *P* = 0.323) correlation between Log_10_ levels of HIV RNA expression in plasma and absolute counts of neutrophils in the genital fluid of the study participants. However, there was a positive and significant correlation (Spearman's Rho = 0.234; *R* ^2^ = 0.028; *Y* = 1.83 + 5.54 − 3∗*X*; *P* = 0.021) between Log_10_ levels of HIV RNA expression in the vagina and absolute counts of neutrophils in the genital fluid of the study participants. There was also a negative but significant association between CD4 count stages and absolute count of neutrophils in the genital fluid: HIV-infected women with CD4 count <200 cells/mm^3^ (*n* = 41) had an average 59.6 ± 6.6 PMN cells/5 high microscopic fields (HMF) as compared to HIV-infected women with CD4 count >200 cells/mm^3^ (*n* = 56) who had an average 18.1 ± 13.8 PMN cells/5HMF (*P* < 0.0001).

Factors associated with symptomatic VVC in HIV infected women are shown in Table 3 using multiple logistic regression. The model showed that the occurrence rate of symptomatic VVC increased with the severity of HIV-induced immunosuppression. Adjusted* odds ratio* values were 9 (*P* = 0.015) and 60 (*P* < 0.0001), respectively, during advanced (CD4+ T cells = 200–349/mm^3^) and severe (CD4+ T cells <200/mm^3^) stages of HIV-induced immunosuppression. In addition, women not receiving HAART were 4-fold more likely (*P* = 0.029) to develop symptomatic VVC than women on HAART. Findings also showed that the probability of developing symptomatic VVC increased with the presence of higher levels of HIV RNA in the blood (approximately 100-fold higher for HIV positive women with plasma HIV RNA load ≥10 000 copies/mL (*P* < 0.0001)). There was a significant negative association between the presence of bacterial vaginosis (Nugent score 7–10) and symptomatic VVC as depicted in Table 3.

In HIV negative women, however, after adjusting for univariate factors that exhibited at least moderate association (*P* < 0.20) with symptomatic VVC (Table 4), only pregnancy was significantly associated with symptomatic VVC (OR = 4.1 95% CI 1.3–10.5, *P* = 0.011).

## 4. Discussion

Findings from this study confirmed previously published reports on symptomatic VVC as being significantly more prevalent among HIV infected than HIV uninfected women [7, 8]. Whilst some studies have shown the association between low CD4+ T-lymphocyte counts and symptomatic VVC [4, 17], our results provide an additional body of evidence that low CD4+ T-lymphocyte counts, particularly CD4+ T cells <200/mm^3^, are significantly associated with symptomatic VVC. In 2006, Beltrame et al. reported that candidal vaginal colonization, a precursor of vaginitis, develops when CD4+ T-lymphocyte counts fall to ≤100 cells/*μ*L in the course of HIV infection [4]. Prior to this report, Duerr et al. published on the rates of candidal vaginal colonization and symptomatic VVC as being similar among nonimmunocompromised HIV-positive women and HIV-negative women [17]. According to these authors, elevated rates of yeast colonization and vaginitis were not observed among their cohort of HIV-infected women before immune compromise. However, rates of vaginal colonization and symptomatic VVC were seen to increase with immune compromise, especially at CD4 counts below 200 cells/mm^3^ [17]. In 2000, Shifrin et al. reported that HIV-positive women with a CD4 count less than 200 cells/mm^3^ had an increase of 8.2 times in the incidence of symptomatic vulvovaginal candidiasis. They suggested that women with low CD4 counts should be closely monitored for the development of symptomatic VVC prior to the development of symptoms independent of candida colonization status [18].

However, in 2003, Ohmit et al. reported that the prevalence of symptomatic VVC, although higher among HIV-infected women, did not significantly differ by HIV serostatus of the participants at baseline [19]. However, the authors found that during follow-up visits, the rate of acquisition of symptomatic VVC was significantly higher among HIV-infected women as compared to HIV-uninfected women. Furthermore, whilst Ohmit et al. identified the level of HIV-associated immunodeficiency (measured by CD4+ T cell counts) as not significantly associated with increased odds for symptomatic VVC, the authors found, however, that odds of symptomatic VVC increased by >2-fold for women whose plasma HIV load was >1000 copies/mL. They found an increase of 11% to 14% for every Log_10_ increase in plasma HIV viral load [19]. Prior to this report, Sobel et al. found out that higher HIV loads rather than lower CD4+ T-lymphocyte counts were associated with statistically significant increased odds for both persistent candidal vaginal colonization and symptomatic VVC [5]. However, the study by Sobel et al. only found an association between plasma HIV viral load and the proportion of* Candida* infections that were non-*C. albicans*, not the absolute prevalence of symptomatic VVC [5].

Data from the present study demonstrated that plasma HIV RNA levels were significantly associated with symptomatic VVC in HIV positive women. Compared with patients whose plasma HIV load was suppressed below the detectable level (<20 copies/mL), cases of symptomatic VVC increased with an increase of plasma HIV load. Plausible biological reasons why blood HIV viral loads can correlate with symptomatic VVC have been published by others. Following a cross-sectional study on oral candidiasis, Gottfredsson et al. hypothesized that high level of plasma HIV-1 can suppress local mucosal immune mechanisms independently of systemic cell-mediated immunity, leading to mucosal colonization with* Candida species* [20]. The authors also speculated that controlling the replication of HIV-1 could possibly restore local mucosal immune functions. Similarly, during an in vitro analysis, Gruber et al. reported that high plasma and therefore salivary or oral HIV-1 load might promote virulence of* C. albicans* at oral mucosal surfaces [21]. In the present study, we found a significant linear correlation between plasma HIV viral load and genital HIV shedding. We also found that HIV viral loads (in plasma and vagina) increased across progressive stages of HIV-induced immunocompromise by CD4+ T cell stages. We can therefore hypothesize that during advanced HIV infection (as measured by low CD4+ T cell levels) with subsequently observed higher HIV viral loads, HIV particles might change the vaginal environment, hence promoting virulence of* Candida species* by switching from its nonpathogenic form into a filamentous form that causes symptomatic VVC. We can further speculate that because ART-naïve HIV-infected women were more likely able to develop symptomatic VVC than women on HAART, controlling the replication of HIV by using HAART could possibly restore local mucosal immune functions in the vagina. This suggests that high levels of plasma HIV load could suppress genital mucosal immune mechanisms independently of systemic cell-mediated immunity, leading to symptomatic VVC. Further studies are required to confirm this hypothesis. Although during our multivariate logistic regression models, genital HIV shedding was not identified as a significant determinant of symptomatic VVC, increased genital HIV load was, however, shown to correlate with symptomatic VVC only in univariate analysis. This could be explained by the fact that HIV load was only measured from the cell-free fraction of cervicovaginal fluid (HIV RNA expression). HIV is more readily detected in the cell-associated component of genital secretions (HIV DNA) than in the cell-free component. It is possible that the cell-associated fraction of vaginal secretions could reflect better shedding of HIV infected cells from the vagina, while the cell-free fraction may only represent HIV released locally by actively replicating cells. Whilst a univariate association was shown between genital HIV load and symptomatic VVC, there was a positive and significant correlation between Log_10_ levels of genital HIV RNA expression and absolute counts of neutrophils in the genital fluid. In addition, due to the inverse relationship between CD4 count and absolute neutrophil count shown in our study, it can be postulated that this is the result of increased HIV shedding in the vagina with a concomitant inflammatory response in the setting of increasing immune suppression. Another study is required to ascertain this hypothesis.

Based on data from a human life challenge model, Fidel Jr. et al. hypothesized that following the interaction of* Candida* with vaginal epithelial cells, symptomatic VVC was associated with signals that promoted a nonprotective inflammatory leukocyte response and concomitant clinical symptoms. They indicated that neutrophils contribute to the pathogenesis and local inflammation in symptomatic VVC [22].

There was a significant negative association between the presence of BV and symptomatic VVC. A high prevalence of BV was diagnosed among both HIV positive and HIV negative women. The rate of BV has been always reported to be very high in the South African province of KwaZulu-Natal. In 1996, Govender et al., during a study on BV and associated pregnancy outcomes among asymptomatic pregnant women attending antenatal clinic at King Edward VIII hospital in Durban/KwaZulu-Natal, reported a prevalence of 52% for BV, and BV was the leading lower genital tract infection according to the authors [23]. In 2002, Moodley et al. during a study at Hlabisa Health District in northern KwaZuluNatal found the prevalence of BV to be 70% among their 598 enrolled women [24]. BV is known as a syndrome characterized by imbalance of the vaginal ecosystem. It can be postulated that in the KwaZulu-Natal province of South Africa, anecdotal reports of the excessive use of topically applied substances in the vagina with antimicrobial effects interferes with the lactobacilli. The depletion of lactobacilli may limit the production of hydrogen peroxide. Vallone et al. postulated that low vaginal pH inhibits CD4 lymphocyte activation and reduces HIV target cells in the vagina, while elevated vaginal pH may enhance HIV adherence to vaginal eukaryotic cells [25]. In this present study, although BV was prevalent among both HIV positive and HIV negative, there was a significant association between BV and higher expression of HIV RNA in the plasma of HIV-infected women. Our findings have also shown a significant association between the composition of the vaginal microbiota and the development of symptomatic VVC in HIV infected women. Using the Nugent score to assess the degree of alteration of the vaginal flora, symptomatic VVC was significantly associated with a score of <7. These findings strongly support the hypothesis that symptomatic VVC is usually associated with a normal vaginal pH (pH < 4.5), while BV is established when pH of the vaginal fluid becomes >4.5. In addition, these findings are also in keeping with results from Moodley et al. who found out that Yeast colonization and symptomatic VVC were inversely related to Nugent's scores [24].

In addition to low Nugent score, another factor that was identified as being significantly associated with symptomatic VVC was pregnancy, particularly among HIV-uninfected women. This finding was consistent with what has been found elsewhere [1].

Although HIV-uninfected women with symptomatic VVC reported a history of antibiotic use within the past 3 months prior to the clinic visit more than women without symptomatic VVC, this difference was not statistically significant. The use of broad spectrum antibiotic has been extensively reported from the literature as a determinant of symptomatic VVC [1]. The fact that patients have been asked if they received any antibiotic in the last 3 months may not provide an accurate information as some patients might forgot or could not differentiate antibiotics from other types of medicines that they received.

Although the presence of concurrent lower genital tract pathogens were not shown to be significantly associated with symptomatic VVC, there was a negative association between the presence of symptomatic VVC and vaginitis caused by* Trichomonas vaginalis*. Moodley et al. reported that* T. vaginalis* was prevalent in patients with all levels of abnormal vaginal flora (bacterial vaginosis score of Gram stain >4) suggesting that this pathogen might contribute to the change in vaginal flora leading to BV [24]. The authors further identified that the presence of yeasts on microscopy was inversely related to the level of ecologic disturbance. They argued that BV environment is not conducive to yeast multiplication, and yeast vaginitis therefore does not occur frequently in the presence of both BV and* T. vaginalis *[24].

## 5. Limitations

A small sample size needs to be underlined as a first limitation. The fact that CD4 counts obtained from the patient medical records might be at least 3 months old presents a limitation in the interpretation of our results, in that the degree of immune suppression may have been over- or underestimated. However, in our study there was a significant negative association between CD4 counts and HIV viral loads in plasma (*P* < 0.0001). A further bias in the study is the fact that we used patients' records of ARV prescriptions and patient interviews as a proxy for ARV treatment adherence. The latter is often subjective. Measurement of drug levels is a more objective test of adherence. However, this was not done in our study.

Although we assessed the history of previous vaginal discharge syndrome, this was not conclusive of symptomatic VVC and hence could not establish possible cases of recurrent VVC. Finally, we prevented colinearities in our logistic models by not including variables that were highly correlated, but the 95% confidence intervals around the odds ratios were slightly wide for certain variables. This might be due to potential variability observed during the measurement of CD4+ T cells and HIV loads. Despite these limitations, findings from this study add on the existing body of evidence on the correlation between HIV-induced immunocompromise and the presence of symptomatic VVC. Commencing HIV-infected women on HAART could prevent new incident cases of symptomatic VVC.

## Figures and Tables

**Table 1:** Baseline characteristics of the study population (*n* = 198) who contributed symptomatic vulvovaginal candidiasis (VVC) and vaginal *Candida species* colonization data by human immunodeficiency virus (HIV) serostatus.

Characteristics	HIV-infected *n* = 97	HIV-uninfected *n* = 101	*P* value
Age in years, median (range)			<0.0001
21 (18–24)	34 (35)	69 (68.3)	
30 (25–34)	48 (49.5)	19 (18.8)	
40.2 (35–46)	15 (15.5)	13 (12.9)	
Major presenting symptoms			0.008
Genital itching and soreness	87 (89.7)	76 (75.3)	
vaginal discharge and low abdominal pain	10 (10.3)	25 (24.7)	
History of antibiotic use within the past 3 months	64 (65.9)	49 (48.5)	0.013
Condom use	25 (25.8)	14 (13.9)	0.035
Pregnancy	4 (4.1)	19 (18.8)	<0.001
History of sexually transmitted infections (STIs) within the past 3 months			0.093
Vaginal discharge syndrome	42 (43.3)	36 (35.6)	
Genital ulcer syndrome	13 (13.4)	7 (6.9)	
No defined sexually transmitted infection	42 (43.3)	58 (57.4)	
Currently isolated pathogens for STIs			0.085
*Trichomonas vaginalis *	15 (15.5)	16 (15.8)	0.683
Chlamydia* *trachomatis	7 (7.2)	4 (3.9)	0.228
Neisseria* *gonorrhoeae	6 (6.2)	5 (4.9)	0.516
*Mycoplasma genitalium *	2 (2.1)	1 (0.9)	0.456
Herpes simplex virus type 2	8 (8.3)	0 (0)	0.999
STI caused by more than 1 aetiology	13 (13.4)	17 (16.8)	0.93
No identified STI pathogen	46 (47.4)	58 (57.4)	1
Vaginal flora (Nugent's score)			0.819
0 to 3	14 (14.4)	14 (13.9)	
4 to 6	8 (8.3)	11 (10.9)	
7 to 10	75 (77.3)	76 (75.3)	
Absolute count of neutrophil cells in genital fluid			0.77
Score 1	42 (43.3)	41 (40.6)	
Score 2	23 (23.7)	21 (20.8)	
Score 3	32 (32.9)	39 (38.6)	
Symptomatic vulvovaginal candidiasis			0.032
Yes	52 (53.6)	38 (37.6)	
No (colonization or absence)	45 (46.4)	63 (62.4)	
CD4+ T cell count (cells/mm^3^)			
<200	41 (42.3)	N/A	N/A
200 to 349	32 (33)	N/A	N/A
≥350	24 (24.7)	N/A	N/A
Plasma HIV viral load (copies/mL)			
≥10 000	22 (22.7)	N/A	N/A
20 to 9999	51 (52.6)	N/A	N/A
<20	24 (24.7)	N/A	N/A
Genital HIV viral load (copies/mL)			
≥10 000	12 (12.4)	N/A	N/A
20 to 9999	21 (21.7)	N/A	N/A
<20	64 (65.9)	N/A	N/A
Users of highly active antiretroviral therapy			
Yes	60 (61.9)	N/A	N/A
No	37 (38.1)	N/A	N/A

N/A: not applicable.

**Table 2:** Univariate associations between variables of interest and symptomatic vulvovaginal candidiasis among HIV-infected women (*n* = 97).

Variables of interest	Symptomatic VVC (*n* = 52)	VVC colonization (*n* = 45)	*P* value
	*n* (%)	*n* (%)	
Age in years, median (range)			0.484
21 (18–24)	21 (40.4)	13 (28.9)	
30 (25–34)	24 (46.2)	24 (53.3)	
40.2 (35–46)	7 (13.5)	8 (17.8)	
Major presenting complains			0.362
Vaginal discharge	4 (7.7)	6 (13.3)	
Genital itching and soreness	48 (92.3)	39 (86.7)	
History of antibiotic use within the past 3 months	36 (69.2)	28 (62.2)	0.467
No history of condom use	42 (80.8)	30 (66.7)	0.113
History of sexually transmitted infections within the past 3 months			0.312
Vaginal discharge syndrome	18 (34.6)	24 (53.3)	
Genital ulcer syndrome	1 (1.9)	1 (2.2)	
Mixed sexually transmitted infections	7 (13.5)	4 (8.9)	
No defined sexually transmitted infections	26 (50)	16 (36)	
Currently isolated pathogens for STIs			0.153
*Trichomonas vaginalis *	5 (9.6)	10 (22.2)	0.07
*Chlamydia trachomatis *	1 (1.9)	6 (13.3)	0.046
*Neisseria gonorrhoeae *	3 (5.8)	3 (6.7)	0.612
*Mycoplasma genitalium *	1 (1.9)	1 (2.2)	0.76
Herpes simplex virus type 2	5 (9.6)	3 (6.7)	0.93
STI caused by more than 1 aetiology	9 (17.3)	4 (8.9)	0.583
No STI pathogen identified	28 (53.8)	18 (40)	1
Vaginal flora (Nugent's scores)			0.01
less than 7 (BV negative)	17 (32.7)	5 (11.1)	
≥7 (BV positive)	35 (67.3)	40 (88.9)	
Pregnancy	4 (7.7)	0 (0)	0.057
Plasma HIV viral load (VL) categories			<0.0001
≥10000 copies	19 (36.5)	3 (6.7)	
20–9999 copies	28 (53.8)	23 (51.1)	
<20 copies	5 (9.6)	19 (42.2)	
Genital HIV viral load (VL) categories			0.002
≥10000 copies	12 (23.1)	0 (0)	
20–9999 copies	12 (23.1)	9 (20)	
<20 copies	28 (53.8)	36 (80)	
CD4+ T cell counts			<0.0001
<200 cells	30 (57.7)	11 (24.4)	
200–349 cells	17 (32.7)	15 (33.3)	
≥350 cells	5 (9.6)	19 (42.2)	
Therapy groups			<0.001
Patients on antiretroviral therapy	26 (50)	34 (75.6)	
Patients not receiving antiretroviral therapy	26 (50)	11 (24.4)	

**Table 3:** Determinants of symptomatic vulvovaginal candidiasis among HIV-infected women.

Independent variables	*B* Coefficient	Standard error	Wald chi-square	OR (95% CI)	*P* value
CD4+ T cell groups					
<200 cells	4.1	0.971	17.847	60.3 (9–134.8)	<0.0001
200–349 cells	2.186	0.901	5.886	8.9 (1.5–17.36)	0.015
≥350 cells			Referent	1	
Vaginal Flora (Nugent's scores)					
less than 7 (BV negative)	3.143	0.954	10.844	23.2 (3.6–50.13)	<0.001
≥7 (BV positive)			Referent	1	
Therapy groups					
Patients not receiving antiretroviral therapy	1.251	0.573	4.768	3.5 (1.1–10.7)	0.029
Patients on antiretroviral therapy			Referent	1	
Constant	−3.13	0.904	11.981		<0.001

Adjusted for previous history of sexually transmitted infections (STIs), presence of current STIs, history of condom use, pregnancy, and presenting symptoms.

**Table 4:** Univariate associations between variables of interest and symptomatic vulvovaginal candidiasis (VVC) among HIV-uninfected women (*n* = 101).

Variables of interest	Symptomatic VVC (*n* = 38)	VVC colonization (*n* = 63)	*P* value
	*n* (%)	*n* (%)	
Age in years, median (range)			0.705
21 (18–24)	26 (68.4)	43 (68.3)	
30 (25–34)	6 (15.8)	13 (20.6)	
40.2 (35–46)	6 (15.8)	7 (11.1)	
History of antibiotic use within the past 3 months	17 (44.7)	32 (50.8)	0.555
No history of condom use	35 (92.1)	52 (82.5)	0.178
History of sexually transmitted infections within the past 3 months			0.504
Vaginal discharge syndrome	14 (36.8)	22 (34.9)	
Genital ulcer syndrome	0 (0)	2 (3.2)	
Mixed sexually transmitted infections	3 (7.9)	2 (3.2)	
No defined sexually transmitted infections	21 (55.3)	37 (58.7)	
Currently isolated pathogens for STIs			0.377
*Trichomonas vaginalis *	5 (13.2)	11 (17.5)	0.333
*Chlamydia trachomatis *	2 (5.3)	2 (3.2)	0.841
*Neisseria gonorrhoeae *	2 (5.3)	3 (4.8)	0.835
*Mycoplasma genitalium *	0 (0)	1 (1.6)	1
Herpes simplex virus type 2	0 (0)	0 (0)	0.053
STI caused by more than 1 aetiology	3 (7.9)	14 (22.2)	1
No STI pathogen identified	26 (68.4)	14 (22.2)	1
Vaginal flora (Nugent's scores)			0.08
Less than 7 (BV negative)	13 (34.2)	12 (19)	
≥7 (BV positive)	25 (65.8)	51 (81)	
Pregnancy	12 (31.6)	7 (11.1)	0.011

BV: bacterial vaginosis.
